# Establishing conservation units to promote recovery of two threatened freshwater mussel species (Bivalvia: Unionida: *Potamilus*)

**DOI:** 10.1002/ece3.7897

**Published:** 2021-07-31

**Authors:** Chase H. Smith, Nathan A. Johnson, Clinton R. Robertson, Robert D. Doyle, Charles R. Randklev

**Affiliations:** ^1^ Department of Integrative Biology University of Texas Austin Texas USA; ^2^ Texas A&M Natural Resources Institute, Texas A&M AgriLife Research Center at Dallas Dallas Texas USA; ^3^ Biology Department Baylor University Waco Texas USA; ^4^ U.S. Geological Survey, Wetland and Aquatic Research Center Gainesville Florida USA; ^5^ Rivers Studies Program Texas Parks and Wildlife Department San Marcos Texas USA

**Keywords:** conservation genetics, DPHS, endangered species, population genomics, recovery

## Abstract

Population genomics has significantly increased our ability to make inferences about microevolutionary processes and demographic histories, which have the potential to improve protection and recovery of imperiled species. Freshwater mussels (Bivalvia: Unionida) represent one of the most imperiled groups of organisms globally. Despite systemic decline of mussel abundance and diversity, studies evaluating spatiotemporal changes in distribution, demographic histories, and ecological factors that threaten long‐term persistence of imperiled species remain lacking. In this study, we use genotype‐by‐sequencing (GBS) and mitochondrial sequence data (mtDNA) to define conservation units (CUs) for two highly imperiled freshwater mussel species, *Potamilus amphichaenus* and *Potamilus streckersoni*. We then synthesize our molecular findings with details from field collections spanning from 1901 to 2019 to further elucidate distributional trends, contemporary status, and other factors that may be contributing to population declines for our focal species. We collected GBS and mtDNA data for individuals of *P. amphichaenus* and *P. streckersoni* from freshwater mussel collections in the Brazos, Neches, Sabine, and Trinity drainages ranging from 2012 to 2019. Molecular analyses resolved disputing number of genetic clusters within *P. amphichaenus* and *P. streckersoni*; however, we find defensible support for four CUs, each corresponding to an independent river basin. Evaluations of historical and recent occurrence data illuminated a generally increasing trend of occurrence in each of the four CUs, which were correlated with recent increases in sampling effort. Taken together, these findings suggest that *P. amphichaenus* and *P. streckersoni* are likely rare throughout their respective ranges. Because of this, the establishment of CUs will facilitate evidence‐based recovery planning and ensure potential captive propagation and translocation efforts are beneficial. Our synthesis represents a case study for conservation genomic assessments in freshwater mussels and provides a model for future studies aimed at recovery planning for these highly imperiled organisms.

## Graphical abstract

Freshwater mussels (Bivalvia: Unionida) represent one of the most imperiled groups of organisms globally. In this study, we couple multiple molecular datasets with survey data to facilitate recovery efforts for two threatened species of freshwater mussels. Our synthesis represents a case study for conservation genomic assessments in freshwater mussels and provides a model for future studies aimed at recovery planning for these highly imperiled organisms.

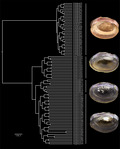

## 1 INTRODUCTION

Population genomics has significantly increased our ability to make inferences about microevolutionary processes (e.g., gene flow, genetic drift, population structure, selection, and mutation) through the use of thousands of genome‐wide markers (Allendorf et al., 2010; Luikart et al., 2003). In comparison with microsatellite or Sanger sequencing methodologies (typically 10–20 markers), genotype‐by‐sequencing (GBS) methods (e.g., restriction enzyme‐based sequencing approaches) have substantially more power to resolve population dynamics given the vast number of molecular markers. Advancements in genomic technologies have been proven useful for improving protection and recovery of imperiled species (Allendorf et al., 2010; Coates et al., 2018; Funk et al., 2012, 2019), even in nonmodel organisms without established genomic resources (Ellegren, 2014).

Conservation biologists and natural resource managers use information on resiliency, redundancy, and representation to inform management efforts for imperiled organisms (Smith, Allan, et al., 2018; Wolf et al., 2015). However, determining “the three Rs” relies on the a priori designation of population units across the species’ range. The U.S. Endangered Species Act (ESA) classifies population units as distinct population segments (USFWS & NMFS, 1996), which are often delineated using natural or man‐made barriers. The rationale for this approach is these structures theoretically promote genetic drift between extant populations. However, molecular studies have shown this may or may not be the case (e.g., Clemento et al., 2009; Grummer & Leaché, 2017; Hoffman et al., 2017), which can have substantial impacts on recovery planning. Thus, it is becoming increasingly evident that the use of molecular data is a more thorough approach to delineate population units for imperiled species. Specifically, the implementation of genomic methodologies to define the boundaries of conservation units (CUs), or population units for conservation (Funk et al., 2012), is a necessity in order to infer the status of populations within imperiled species (e.g., genetic diversity, population demographics, population trends).

Freshwater mussels (Bivalvia: Unionida) are a group of aquatic bivalves comprised of approximately 840 species (Graf & Cummings, 2007), but also represent one of the most imperiled groups globally (Lopes‐Lima et al., 2018). Widespread alteration to freshwater ecosystems has led to systemic declines in abundance of both common and rare freshwater mussel species (Haag & Williams, 2014; Vaughn & Taylor, 1999). These declines stem from a suite of traits that make freshwater mussels sensitive to ecosystem state change, including filter feeding, limited locomotive capabilities, and an obligate parasitic life cycle that requires codependence with hosts to complete metamorphosis (Haag, 2012; Randklev et al., 2019; Williams et al., 1993). Widespread decline has major ecological ramifications considering the loss of freshwater mussel biodiversity can negatively impact ecosystem function of freshwater systems (Vaughn, 2018; Vaughn et al., 2008). These factors have led conservation biologists and natural resource managers to prioritize freshwater mussels as a group of greatest conservation concern and a keystone for guiding freshwater ecosystem restoration (Ferreira‐Rodríguez et al., 2019; Haag & Williams, 2014; Lopes‐Lima et al., 2018; Williams et al., 1993).

Conservation and management of freshwater mussels is largely based on geography (e.g., USFWS, 1989, 1996, 2000, 2010), and there remains a need to incorporate molecular data into conservation and recovery planning (e.g., Ferreira‐Rodríguez et al., 2019; McMurray & Roe, 2017). Many freshwater mussel species have been shown to depict significant genetic structure (Elderkin et al., 2008; Grobler et al., 2006; Inoue et al., 2015; Johnson et al., 2018; Scott et al., 2020; Smith & Johnson, 2020; Smith et al., 2019; Smith, Johnson, et al., 2018; Zanatta & Wilson, 2011), but the recognition of CUs within species remains rare (Grobler et al., 2006; Smith & Johnson, 2020). Although CUs (i.e., genetically distinct population units) for invertebrates are not afforded protection under the ESA (Waples et al., 2013), the establishment of CUs has been integral in the development of species status assessments, effective genetic management, and species recovery of aquatic species (e.g., Avise, 2004; Laikre et al., 2005). Therefore, using genetic information (e.g., genetic diversity, population structure) to guide status assessments represents a powerful approach for freshwater mussel conservation.

In this study, we use molecular data and available survey information to delineate CUs and investigate distributional trends of two highly imperiled freshwater mussel species: *Potamilus amphichaenus* and *Potamilus streckersoni*. *Potamilus amphichaenus* is endemic to the Sabine, Neches, and Trinity River drainages in eastern Texas (Howells et al., 1996), while the distribution for *P. streckersoni* is restricted to the Brazos River drainage in central Texas (Smith et al., 2019). As members of *Potamilus*, both species are presumed to be host specialists, with glochidia only transforming on *Aplodinotus grunniens* (Bosman et al., 2014; Smith et al., 2020), a common molluscivorous fish distributed throughout Gulf of Mexico drainages (Page & Burr, 2011). Both of these species are listed as threatened in the state of Texas (TPWD, 2020), and *P. amphichaenus* is currently under review for listing under the ESA (USFWS, 2009). We set out to accomplish four objectives in this study to better inform conservation and recovery practices: (a) estimate genetic diversity and identify genetic structure throughout the range of *P. amphichaenus* and *P. streckersoni* using GBS and mitochondrial DNA (mtDNA) data, (b) use available survey information to evaluate distributional trends over time and contemporary status of *P. amphichaenus* and *P. streckersoni*, (c) delineate conservation units throughout the range of *P. amphichaenus* and *P. streckersoni*, and (4) discuss our findings in terms of their impact on future conservation, management, and recovery practices.

## 2 MATERIALS AND METHODS

### 2.1 Sampling design and DNA extraction

We sampled *P. amphichaenus* and *P. streckersoni* from focal drainages in Texas (i.e., Brazos, Neches, Sabine, and Trinity). *Potamilus amphichaenus* was collected from 24 localities (Neches = 8, Sabine = 8, Trinity = 8) and *P. streckersoni* was collected from 22 localities in the Brazos River drainage (Table 1). Outgroups were not included considering multiple phylogenetic studies have resolved *P. amphichaenus* and *P. streckersoni* as sister species with strong support (Smith et al., 2019, 2020). Genomic DNA was extracted from fresh mantle clips using the Gentra PureGene extraction kit following manufacturer protocol (Qiagen; Hilden, Germany). High molecular weight DNA was ensured by visualizing isolations on a 1% agarose gel stained with GelRed Nucleic Acid Stain (Biotium), and the purity of each isolation was quantified using a NanoDrop™ (Thermo Fisher Scientific).

**TABLE 1:** Molecular material examined in this study

Taxon	ID	Source	Drainage	ND1 Accession	SRA Accession
*Potamilus amphichaenus*	PampNec009	UF438920	Neches	MK045161	SAMN16131508
*Potamilus amphichaenus*	PampNec021	JBFMC8043.2	Neches	MK045162	
*Potamilus amphichaenus*	PampNec022	JBFMC8043.3	Neches	MK045163	
*Potamilus amphichaenus*	PampNec023	JBFMC8043.4	Neches	MK045164	
*Potamilus amphichaenus*	PampNec046	JBFMC9500.1	Neches	MW001718	SAMN16131509
*Potamilus amphichaenus*	PampNec047	JBFMC9500.2	Neches	MW001719	
*Potamilus amphichaenus*	PampNec048	JBFMC9500.3	Neches	MW001720	SAMN16131510
*Potamilus amphichaenus*	PampNec049	JBFMC9500.4	Neches	MW001721	SAMN16131511
*Potamilus amphichaenus*	PampNec050	JBFMC9500.5	Neches	MW001722	SAMN16131512
*Potamilus amphichaenus*	PampNec051	JBFMC9500.6	Neches	MW001723	SAMN16131513
*Potamilus amphichaenus*	PampNec052	JBFMC9500.7	Neches	MW001724	
*Potamilus amphichaenus*	PampNec053	JBFMC9500.8	Neches	MW001725	SAMN16131514
*Potamilus amphichaenus*	PampNec054	JBFMC9500.9	Neches	MW001726	SAMN16131515
*Potamilus amphichaenus*	PampNec055	JBFMC9500.10	Neches	MW001727	SAMN16131516
*Potamilus amphichaenus*	PampNec056	JBFMC9510.1	Neches	MW001728	SAMN16131517
*Potamilus amphichaenus*	PampNec057	JBFMC9517.1	Neches	MW001729	SAMN16131518
*Potamilus amphichaenus*	PampNec058	JBFMC9517.2	Neches	MW001730	SAMN16131519
*Potamilus amphichaenus*	PampNec059	JBFMC9517.3	Neches	MW001731	SAMN16131520
*Potamilus amphichaenus*	PampNec060	JBFMC9517.4	Neches	MW001732	SAMN16131521
*Potamilus amphichaenus*	PampNec061	JBFMC9519.1	Neches	MW001733	SAMN16131522
*Potamilus amphichaenus*	PampNec062	JBFMC9519.2	Neches	MW001734	SAMN16131523
*Potamilus amphichaenus*	PampNec063	JBFMC9519.3	Neches	MW001735	SAMN16131524
*Potamilus amphichaenus*	PampNec064	JBFMC9519.4	Neches	MW001736	SAMN16131525
*Potamilus amphichaenus*	PampNec065	JBFMC9519.5	Neches	MW001737	SAMN16131526
*Potamilus amphichaenus*	PampNec066	JBFMC9519.6	Neches	MW001738	SAMN16131527
*Potamilus amphichaenus*	PampNec067	JBFMC9519.7	Neches	MW001739	SAMN16131528
*Potamilus amphichaenus*	PampNec068	JBFMC9519.8	Neches	MW001740	SAMN16131529
*Potamilus amphichaenus*	PampNec069	JBFMC9519.9	Neches	MW001741	SAMN16131530
*Potamilus amphichaenus*	PampNec070	JBFMC9519.10	Neches	MW001742	SAMN16131531
*Potamilus amphichaenus*	PampNec071	JBFMC9519.11	Neches	MW001743	SAMN16131532
*Potamilus amphichaenus*	PampNec072	JBFMC9519.12	Neches	MW001744	SAMN16131533
*Potamilus amphichaenus*	PampNec078	JBFMC9572.1	Neches	MW001750	SAMN16131534
*Potamilus amphichaenus*	PampNec079	JBFMC9571.1	Neches	MW001751	SAMN16131535
*Potamilus amphichaenus*	PampNec080	JBFMC9571.2	Neches	MW001752	SAMN16131536
*Potamilus amphichaenus*	PampNec081	JBFMC9571.3	Neches	MW001753	SAMN16131537
*Potamilus amphichaenus*	PampNec082	JBFMC9571.4	Neches	MW001754	SAMN16131538
*Potamilus amphichaenus*	PampNec083	JBFMC9571.5	Neches	MW001755	SAMN16131539
*Potamilus amphichaenus*	PampNec084	JBFMC9571.6	Neches	MW001756	SAMN16131540
*Potamilus amphichaenus*	PampSab018	UF439482.237	Sabine	MK045101	SAMN16131541
*Potamilus amphichaenus*	PampSab019	UF439483.238	Sabine	MK045165	SAMN16131542
*Potamilus amphichaenus*	PampSab044	JBFMC8634.1	Sabine	MW001700	SAMN16131543
*Potamilus amphichaenus*	PampSab045	JBFMC8699	Sabine	MW001701	SAMN16131544
*Potamilus amphichaenus*	PampSab073	JBFMC9526.1	Sabine	MW001745	SAMN16131545
*Potamilus amphichaenus*	PampSab074	JBFMC9526.2	Sabine	MW001746	SAMN16131546
*Potamilus amphichaenus*	PampSab075	JBFMC9529.1	Sabine	MW001747	SAMN16131547
*Potamilus amphichaenus*	PampSab076	JBFMC9531.1	Sabine	MW001748	SAMN16131548
*Potamilus amphichaenus*	PampSab077	JBFMC9532.1	Sabine	MW001749	SAMN16131549
*Potamilus amphichaenus*	PampTri010	UF438957	Trinity	MK045166	
*Potamilus amphichaenus*	PampTri011	N/A	Trinity	MK045167	
*Potamilus amphichaenus*	PampTri012	N/A	Trinity	MK045168	
*Potamilus amphichaenus*	PampTri013	N/A	Trinity	MK045099	
*Potamilus amphichaenus*	PampTri015	UF439095	Trinity	MK045100	
*Potamilus amphichaenus*	PampTri016	UF439095	Trinity	MK045169	
*Potamilus amphichaenus*	PampTri017	UA2997	Trinity	MK045170	
*Potamilus amphichaenus*	PampTri027	JBFMC8442.1	Trinity	MK045171	SAMN16131550
*Potamilus amphichaenus*	PampTri028	JBFMC8442.2	Trinity	MK045172	SAMN16131551
*Potamilus amphichaenus*	PampTri029	JBFMC8442.3	Trinity	MK045173	SAMN16131552
*Potamilus amphichaenus*	PampTri030	JBFMC8442.4	Trinity	MK045174	SAMN16131553
*Potamilus amphichaenus*	PampTri031	JBFMC8442.5	Trinity	MK045175	SAMN16131554
*Potamilus amphichaenus*	PampTri032	JBFMC8442.6	Trinity	MK045176	SAMN16131555
*Potamilus amphichaenus*	PampTri033	JBFMC8444.1	Trinity	MK045177	
*Potamilus amphichaenus*	PampTri034	JBFMC8444.2	Trinity	MK045178	SAMN16131556
*Potamilus amphichaenus*	PampTri035	JBFMC8444.3	Trinity	MK045179	SAMN16131557
*Potamilus amphichaenus*	PampTri036	JBFMC8444.4	Trinity	MK045180	SAMN16131558
*Potamilus amphichaenus*	PampTri037	JBFMC8444.5	Trinity	MK045181	SAMN16131559
*Potamilus amphichaenus*	PampTri038	JBFMC8444.6	Trinity	MK045182	SAMN16131560
*Potamilus amphichaenus*	PampTri039	JBFMC8450.1	Trinity	MK045183	SAMN16131561
*Potamilus amphichaenus*	PampTri041	JBFMC8450.3	Trinity	MK045184	SAMN16131562
*Potamilus amphichaenus*	PampTri042	JBFMC8450.4	Trinity	MK045185	SAMN16131563
*Potamilus amphichaenus*	PampTri043	JBFMC8468.1	Trinity	MK045186	SAMN16131564
*Potamilus streckersoni*	PohiBra034	UF439475.019	Brazos	MK045134	SAMN16131570
*Potamilus streckersoni*	PohiBra035	UF439476.020	Brazos	MK045135	SAMN16131571
*Potamilus streckersoni*	PohiBra036	UF439477.021	Brazos	MK045094	
*Potamilus streckersoni*	PohiBra001	UF441294	Brazos	MK045095	SAMN16131565
*Potamilus streckersoni*	PohiBra002	UF441294	Brazos	MK045136	SAMN16131566
*Potamilus streckersoni*	PohiBra003	UF441294	Brazos	MK045137	SAMN16131567
*Potamilus streckersoni*	PohiBra004	UF441294	Brazos	MK045096	SAMN16131568
*Potamilus streckersoni*	PohiBra005	UF438262	Brazos	MK045138	SAMN16131569
*Potamilus streckersoni*	PohiBra037	UF439478.169	Brazos	MK045139	SAMN16131572
*Potamilus streckersoni*	PohiBra038	UF439478.170	Brazos	MK045140	SAMN16131573
*Potamilus streckersoni*	PohiBra039	UF439478.171	Brazos	MK045141	SAMN16131574
*Potamilus streckersoni*	PohiBra040	UF439478.172	Brazos	MK045142	SAMN16131575
*Potamilus streckersoni*	PohiBra041	UF439478.173	Brazos	MK045143	SAMN16131576
*Potamilus streckersoni*	PohiBra042	UF439479.216	Brazos	MK045144	SAMN16131577
*Potamilus streckersoni*	PohiBra043	UF439480.220	Brazos	MK045145	SAMN16131578
*Potamilus streckersoni*	PohiBra044	UF439481.231	Brazos	MK045146	SAMN16131579
*Potamilus streckersoni*	PohiBra045	UF439481.232	Brazos	MK045147	SAMN16131580
*Potamilus streckersoni*	PohiBra046	JBFMC8176.1	Brazos	MK045148	
*Potamilus streckersoni*	PohiBra049	JBFMC8402.2	Brazos	MK045149	
*Potamilus streckersoni*	PohiBra051	JBFMC8402.4	Brazos	MK045150	
*Potamilus streckersoni*	PohiBra052	JBFMC8402.5	Brazos	MK045151	
*Potamilus streckersoni*	PohiBra053	JBFMC8402.6	Brazos	MK045152	
*Potamilus streckersoni*	PohiBra054	JBFMC8406.1	Brazos	MK045153	
*Potamilus streckersoni*	PohiBra055	JBFMC8406.2	Brazos	MK045154	
*Potamilus streckersoni*	PohiBra057	JBFMC8411.1	Brazos	MK045155	
*Potamilus streckersoni*	PohiBra058	JBFMC8411.2	Brazos	MK045156	
*Potamilus streckersoni*	PohiBra059	JBFMC8433.1	Brazos	MK045157	
*Potamilus streckersoni*	PohiBra061	JBFMC8492.2	Brazos	MK045158	SAMN16131581
*Potamilus streckersoni*	PohiBra062	JBFMC8492.3	Brazos	MK045159	SAMN16131582
*Potamilus streckersoni*	PohiBra063	JBFMC8492.4	Brazos	MK045160	SAMN16131583
*Potamilus streckersoni*	PohiBra064	UF439526	Brazos	MW001702	SAMN16131584
*Potamilus streckersoni*	PohiBra065	UF439526	Brazos	MW001703	SAMN16131585
*Potamilus streckersoni*	PohiBra066	UF439526	Brazos	MW001704	SAMN16131586
*Potamilus streckersoni*	PohiBra067	UF439526	Brazos	MW001705	SAMN16131587
*Potamilus streckersoni*	PohiBra068	UF439526	Brazos	MW001706	SAMN16131588
*Potamilus streckersoni*	PohiBra069	UF439526	Brazos	MW001707	
*Potamilus streckersoni*	PohiBra070	JBFMC9542.1	Brazos	MW001708	SAMN16131589
*Potamilus streckersoni*	PohiBra071	UF439535	Brazos	MW001709	SAMN16131590
*Potamilus streckersoni*	PohiBra072	UF439536	Brazos	MW001710	SAMN16131591
*Potamilus streckersoni*	PohiBra073	UF439536	Brazos	MW001711	SAMN16131592
*Potamilus streckersoni*	PohiBra074	UF439536	Brazos	MW001712	SAMN16131593
*Potamilus streckersoni*	PohiBra075	UF439536	Brazos	MW001713	SAMN16131594
*Potamilus streckersoni*	PstrBra076	UF439537	Brazos	MW001714	SAMN16131595
*Potamilus streckersoni*	PstrBra077	UF439537	Brazos	MW001715	SAMN16131596
*Potamilus streckersoni*	PstrBra078	UF439538	Brazos	MW001716	SAMN16131597
*Potamilus streckersoni*	PstrBra079	UF439538	Brazos	MW001717	SAMN16131598

Note

Museum catalog numbers, GenBank accession numbers, and SRA sample accession numbers for each individual are provided. Museum abbreviations are as follows: JBFMC, Joseph Britton Freshwater Mussel Collection, UA, Alabama Museum of Natural History, and UF, Florida Museum.

### 2.2 Library preparation, sequencing, and filtering

Sequencing for SNP genotyping was performed using DArTseq™ (DArT Pty Ltd). Briefly, DNA samples were processed in digestion and ligation reactions using the *Pstl*‐*Sphl* restriction enzyme combination following Kilian et al. (2012) but replacing a single *Pstl*‐compatible adaptor with two different adaptors corresponding to each restriction enzyme. The *Pstl*‐compatible adapter was designed to include the Illumina flow cell attachment sequence and the reverse adapter contained the flow cell attachment region and *Sphl*‐compatible attachment sequence (see Elshire et al., 2011). Fragments were amplified using the following thermal cycling conditions: 1‐min initial denaturation at 94°C, then 30 cycles of denaturation (20 s, 94°C), annealing (30 s, 58°C), and extension (45 s, 72°C), with a final extension of 7 min at 72°C. The resulting products were subsequently sequenced using 75 base pair (bp) single‐end sequencing on an Illumina Hiseq‐2500.

Reads were demultiplexed using the individual‐specific barcode sequence ligated to the samples. Reads were then processed using proprietary DArT Pty Ltd analytical pipelines (see Georges et al., 2018; Wenzl et al., 2004). At first pass, poor quality reads were filtered (barcode Phred score < 30, read Phred score < 10). Retained sequences were truncated to 69 bp and aggregated using the DArT fast clustering algorithm with a Hamming distance threshold of 3 bp. Error correction was performed using a proprietary algorithm which corrects low‐quality bases (Phred score < 20) with a corresponding high‐quality singleton tag (Phred score > 25). Identical sequences were then collapsed, and SNPs were called using DArTsoft14.

Additional filtering was performed in the dartR package v 1.1.11 (Gruber et al., 2018) using R v 3.5.1 (R Core Team, 2018) following similar methodologies as Georges et al. (2018). Loci with less than 100% reproducibility, a statistic that measures the concordance of a genotype between a minimum of 27 (~30% of genotyped individuals) technical replicates (see Wenzl et al., 2004), and greater than 30% missing data were removed. Subsequently, individuals with greater than 30% missing data and minor allele frequencies (<0.05) were removed from the dataset. We then removed secondary SNPs by retaining the SNP with the highest degree of polymorphism at each locus. Lastly, we excluded loci found to be in linkage disequilibrium using the R package SNPRelate v 1.16.0 (Zheng et al., 2012).

### 2.3 Genotype‐by‐sequencing analyses

A total of 91 individuals of *P. amphichaenus* (Sabine = 9, Neches = 33, Trinity = 15) and *P. streckersoni* (Brazos = 34) were used in all GBS analyses (Table 1). Phylogenomic inference was performed on a concatenated alignment of all full loci using BEAST v 2.6.2 (Bouckaert et al., 2014). Before the analysis, we used bModelTest v 1.2.1 (Bouckaert & Drummond, 2017) to estimate the best nucleotide substitution model for the analysis using the “transitionTransversionsplit” set of models. A strict molecular clock was paired with a GTR + I + G model of nucleotide substitution. The analysis consisted of 10^9^ MCMC generations logging every 1,000 trees with an initial 50% burn‐in. Tracer v 1.7 (Rambaut et al., 2018) was used to evaluate the trace log to ensure proper burn‐in and convergence of all parameters (ESS > 200). A maximum clade credibility tree was estimated in TREEANNOTATOR v 2.6.0 (Bouckaert et al., 2014).

For all downstream GBS analyses, individuals were binned into four groups based on drainage of capture: (a) *P. amphichaenus* from the Sabine, *P. amphichaenus* from the Neches, *P. amphichaenus* from the Trinity, and *P. streckersoni* from the Brazos. Estimates of genetic diversity and population substructuring were conducted using a custom R script utilizing numerous packages (available at https://github.com/chasesmith15/Potamilus_EE). We calculated allelic richness (AR), observed heterozygosity (*H*_o_), expected heterozygosity (*H*_e_), and inbreeding coefficient (*F*_IS_) for each group using diveRsity v 1.9.90 (Keenan et al., 2013). For AR and *F*_IS_, 999 bootstrap replicates were used with a critical value of 0.05. Fixed alleles and private alleles for each group were determined in dartR. To visualize genetic structure data relative to geographical distribution, we performed a principal coordinate analysis (PCoA) in dartR. We also performed a discriminant analysis of principal components (DAPC) in adegenet v 2.1.0 (Jombart, 2008; Jombart & Ahmed, 2011) on the first two PCs and DA eigenvalues. Additionally, DAPC predicts group membership probability for each sample, and we compared clustering results to membership designations as stated above. The best‐fit number of clusters (*K*) was determined using *k*‐means.

We used traditional and model‐based methodologies to identify patterns of population structure in our genomic data. First, we calculated pairwise *F*_ST_ values using the R package StAMPP v 1.5.1 (Pembleton et al., 2013) and implemented 999 bootstrap replicates. We also used the Bayesian clustering algorithm fastSTRUCTURE (Raj et al., 2014) and the non‐negative matrix factorization algorithm TESS3 (Caye et al., 2016). Briefly, fastSTRUCTURE uses the same algorithm as the program STRUCTURE (Pritchard et al., 2000) but is designed for large SNP datasets to reduce computational demand. To account for the possibility of interspecific signal masking intraspecific population structure in model‐based approaches, we ran three datasets in fastSTRUCTURE and TESS3 analyses: (a) all individuals, (b) *P. amphichaenus* only (*n* = 57), and (c) *P. streckersoni* only (*n* = 34). Monomorphic loci and loci under linkage disequilibrium were filtered after creating species‐specific datasets. We modeled *K* = 1–10 to assess population genetic structure in each dataset, and the chooseK.py script in fastSTRUCTURE was used to select the best *K* value to explain structure and maximize likelihood. TESS3 uses ancestry proportions distributed over geographic space, and distinct clines are estimated from both genetic and geographic data (Caye et al., 2016). We incorporated collection localities with our genotypic data and modeled *K* = 1–10 for each dataset using TESS3. Cross‐validation criterion was used to select the most likely K. The geographic coverage of each group (*K* = 4) was modeled using TESS3, and we illustrated the distribution of genetic variation at the hydrological unit code (HUC) 8‐level.

To test the marginal likelihood of differing clustering scenarios, we used the coalescent‐based model SNAPP v 1.5.0 (Bryant et al., 2012) as implemented in BEAST. Given the computational demand of SNAPP, we used the 100 SNPs with the highest degree of polymorphism in our dataset. We estimated the marginal likelihood for three clustering scenarios (*K* = 2–4): (a) *P. amphichaenus* and *P. streckersoni*; (b) *P. amphichaenus* from the Neches + Sabine, *P. amphichaenus* from the Trinity, and *P. streckersoni*; and (c) *P. amphichaenus* from the Neches, *P. amphichaenus* from the Sabine, *P. amphichaenus* from the Trinity, and *P. streckersoni*. Each estimation was conducted using path sampling with 48 steps and 100,000 MCMC steps with a 10,000 preburn‐in (Leaché et al., 2014). Bayes factor delimitation (BFD) was used to assess support for each of the clustering scenarios with 2lnBF > 10 representing significant support (Kass & Raftery, 1995).

We used neestimator v 2.1 (Do et al., 2014) to estimate effective population size (*N_e_*) and the number of effective breeders (*N_b_*) for the four groups based on drainage of capture. The molecular coancestry (Nomura, 2008) and linkage disequilibrium (Waples, 2006) methods were used to estimate *N_b_* and *N_e_*, respectively. Per developers' recommendations, a critical value of 0.05, singleton exclusion, and jackknifed confidence intervals were used to reduce inflated estimates and address potential linkage.

### 2.4 Mitochondrial data generation and analyses

We amplified and sequenced the mitochondrial (mtDNA) gene *NADH dehydrogenase subunit 1* (ND1) to assess genetic variation in the mtDNA genome. Primers used for PCR and sequencing were ND1 5′‐TGGCAGAAAAGTGCATCAGATTAAAGC‐3′ and 5′‐CCTGCTTGGAAGGCAAGTGTACT‐3′ (Serb et al., 2003). PCR amplifications were performed in a 25 µl mixture of molecular grade water (8.5 µl), MyTaq™ Red Mix (12.5 µl; Bioline), primers (1.0 µl each), and DNA template (50 ng). Thermal cycling conditions followed the Serb et al. (2003). PCR products were sent to the Molecular Cloning Laboratories (MCLAB, South San Francisco, CA) for bidirectional sequencing on an ABI 3730.

A total of 116 individuals of *P. amphichaenus* (Sabine = 9, Neches = 38, Trinity = 23) and *P. streckersoni* (Brazos = 46) were used in all mtDNA analyses (Table 1). Phylogenetic inference was performed on the ND1 alignment using BEAST. Before the analysis, we used bModelTest to estimate nucleotide substitution models for each codon position using the “transitionTransversionsplit” set of models. A strict molecular clock was specified for each codon position for both bModelTest and the standard BEAST analysis, and the analyses consisted of 5 * 10^7^ and 10^8^ MCMC generations, respectively. Tracer was used to ensure convergence of all parameters for both analyses, and a maximum clade credibility tree was estimated in TREEANNOTATOR.

For all subsequent mtDNA analyses, individuals were binned into four groups based on drainage of capture: (a) *P. amphichaenus* from the Sabine, *P. amphichaenus* from the Neches, *P. amphichaenus* from the Trinity, and *P. streckersoni* from the Brazos. We used DnaSP v 6.12 (Rozas et al., 2017) to estimate unique haplotypes (*h*), haplotype diversity (Hd), mean number of nucleotide differences (*k*), and mean nucleotide diversity (*π*) for the four groups used in GBS analyses. We calculated pairwise *F*_ST_ values and conducted an AMOVA in Arlequin v 3.5.2.2 (Excoffier & Lischer, 2010) to analyze differentiation between populations of *P. amphichaenus* and *P. streckersoni*. Estimations of *F*_ST_ were conducted using 1,000 bootstrap replicates. To visualize genetic differentiation with respect to geographic distribution, a haplotype network was generated for *P. amphichaenus* and *P. streckersoni* using a median‐joining network in PopART 1.7 (Leigh & Bryant, 2015) with the default epsilon value set at 0 (Bandelt et al., 1999). Complete deletion was used for missing data at any given nucleotide position.

### 2.5 Distribution and abundance estimates

To estimate relative abundance and distributional trends of *P. amphichaenus* and *P. streckersoni* throughout their known ranges, we compiled available or generated data from freshwater mussel surveys that detected live specimens in the Brazos, Neches, Sabine, and Trinity River drainages (Ford et al., ,^2009^, ^2014^, ^2016^; Randklev et al., 2011, 2017, 2020; Smith et al., 2019). For select sites where survey effort was reported (e.g., survey time, number of surveyors), we estimated abundance using catch per unit effort (CPUE), which is calculated by dividing the total number of live individuals by the total person‐hours. Resulting estimates of CPUE were plotted to visualize distribution and relative abundance of both species.

In addition to assessing relative abundance for our focal species, we evaluated spatiotemporal changes in distribution for *P. amphichaenus* and *P. streckersoni* following Buckwalter et al. (2018) with modifications to accommodate our dataset. This approach entailed enumerating the number of detections per HUC sampled (DPHS) for each species per the following eight a priori designated time periods: (a) 1901–1972, (b) 1973–1983, (c) 1984–1994, (d) 1995–1998, (e) 1999–2003, (f) 2004–2009, (g) 2010–2012, and (h) 2013–2019. These eight time periods were chosen because they encompass more than 50% of HUCs within the presumptive range of *P. amphichaenus* and *P. streckersoni*. We then calculated DPHS by dividing the total number of HUCs in which the species was detected by the total number of HUCs sampled throughout the species range, per time period. Only live and fresh dead records were considered as detections for DPHS estimates because time of death for long‐dead shell cannot reliably be determined. We generated scatter plots and fit a linear regression for each species to visualize DPHS with respect to time in the R package ggplot2 (Wickham, 2016).

Given the recent increase in freshwater mussel surveys throughout the range of *P. amphichaenus* and *P. streckersoni*, we compared the relationship between the total number of surveys and DPHS estimates over time to test whether distributional trends were significantly influenced by survey effort. To do this, the total number of surveys performed within the 8 time periods was compiled and subsequently log‐transformed to improve linearity. We then calculated the correlation between DPHS and survey effort in the R core package stats. To test the null hypothesis that DPHS was not influenced by survey effort, we fit a linear model to DPHS with respect to survey effort and assessed statistical significance using an ANOVA in the R package car (Fox & Weisberg, 2019).

## 3 RESULTS

### 3.1 Genotype‐by‐sequencing analyses

**FIGURE 1:**
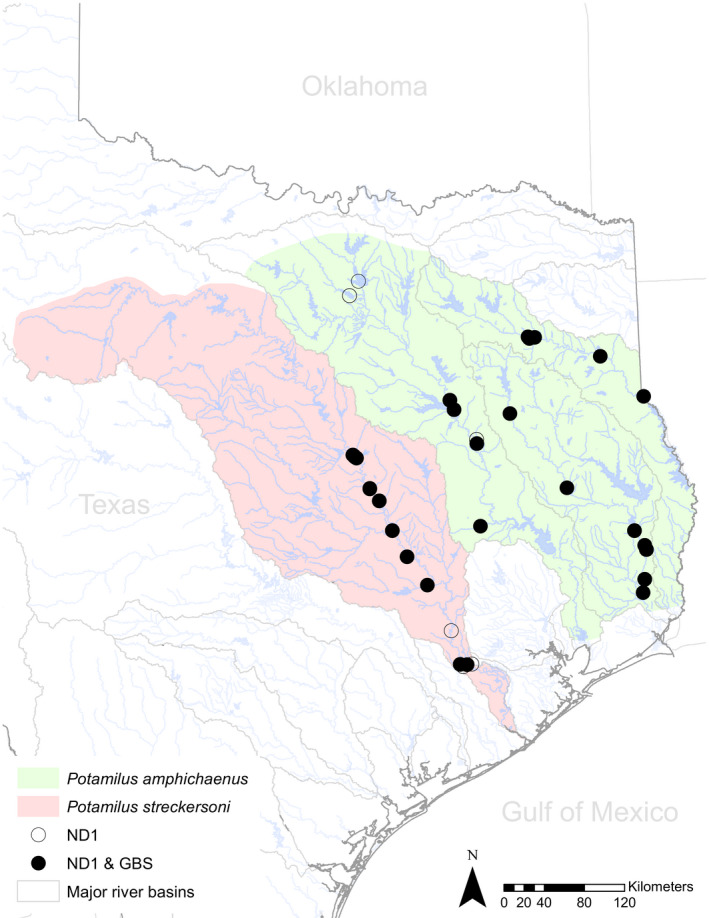
Collection localities for individuals of *Potamilus amphichaenus* and *Potamilus streckersoni* used in molecular analyses. Green and red shading represents the hypothetical historical distributions for *P. amphichaenus* and *P. streckersoni*, respectively. Open circles represent individuals used only in ND1 analyses, and filled circles represent individuals used in both GBS and ND1 analyses

**FIGURE 2:**
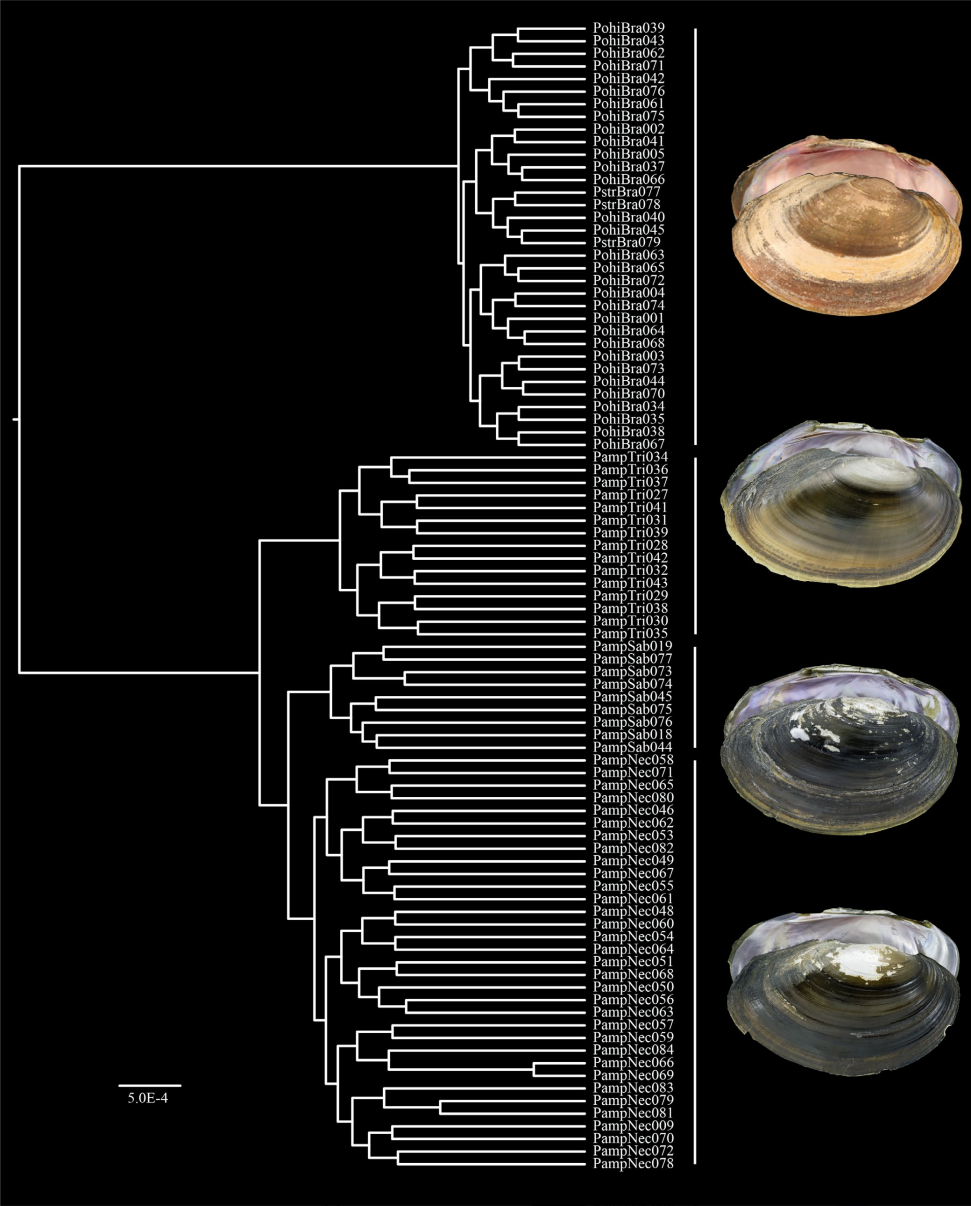
Phylogenetic reconstruction of *Potamilus amphichaenus* and *Potamilus streckersoni* based on genotype‐by‐sequencing data. All nodes representing relationships between drainages (i.e., *P. amphichaenus* from the Neches, Sabine, and Trinity; and *P. streckersoni*) had full posterior probability support (PP = 1.0). From the top, specimen voucher numbers are as follows: *P. streckersoni*—UF439497, *P. amphichaenus* from the Trinity—JBFMC8450.1, *P. amphichaenus* from the Sabine—JBFMC8634.1, and *P. amphichaenus* from the Neches—JBFMC8043.1

A total of 91 individuals of *P. amphichaenus* (57) and *P. streckersoni* (34) were sequenced, and 14,142 of the total 65,465 polymorphic loci were retained after filtering (Figure 1; Table 1; dataset and script available at https://github.com/chasesmith15/Potamilus_EE). Raw reads are deposited in the SRA (BioProject ID: PRJNA663379), and sample accession numbers can be found in Table 1. A GTR + I + G model of nucleotide evolution was selected by bModelTest and the topological reconstruction generated by BEAST resolved four strongly supported (PP = 1.0) clades representing *P. amphichaenus* from the Sabine, *P. amphichaenus* from the Neches, *P. amphichaenus* from the Trinity, and *P. streckersoni* (Figure 2). Convergence of the analysis was supported by ESS values for each parameter greater than 200, and a 50% burn‐in was deemed appropriate for by Tracer.

**FIGURE 3:**
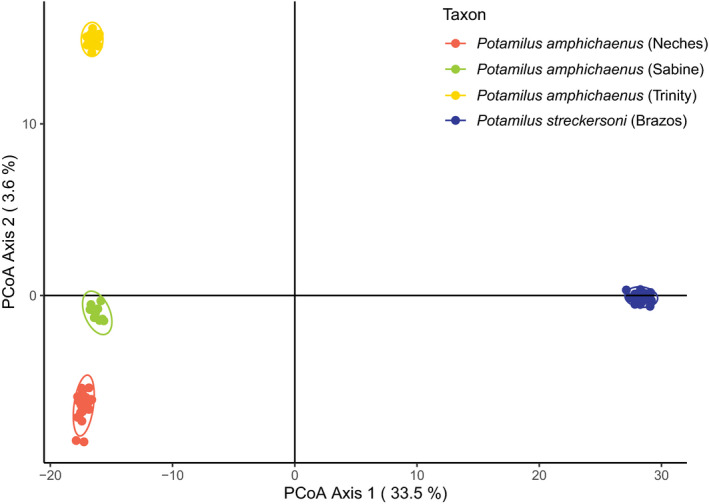
PCoA of *Potamilus amphichaenus* and *Potamilus streckersoni* based on genotype‐by‐sequencing data. Colors correspond to the following groupings: *P. amphichaenus* from the Neches—red, *P. amphichaenus* from the Sabine—green, *P. amphichaenus* from the Trinity—yellow, and *P. streckersoni*—blue. Ellipses represent the 95% confidence interval for each group

Estimates for AR, *H*_o_, *H*_e_, and *F*_IS_ for each group are reported in Table 2. AR, *H*_o_, and *H*_e_ were much lower in *P. streckersoni*; however, *F*_IS_ was also lower in *P. streckersoni* than populations of *P. amphichaenus*. Fixed and private alleles were much higher in comparisons of *P. streckersoni* and populations of *P. amphichaenus*, and fixation was low across populations of *P. amphichaenus* (Table 2). PCoA showed 4 distinct groupings: *P. amphichaenus* from the Sabine, *P. amphichaenus* from the Neches, *P. amphichaenus* from the Trinity, and *P. streckersoni* (Figure 3). The first axis defined 33.5% of the variance (*P. amphichaenus* from *P. streckersoni*), and the second axis described 3.6% of the variance (populations of *P. amphichaenus*). *k*‐means supported the best number of clusters as *K* = 4, aligning with groupings depicted by PCoA. All clusters were strongly supported by DAPC, with all individuals having 100% membership probability when compared to designations by drainage.

**TABLE 2:** Summary of genetic diversity statistics for *Potamilus amphichaenus* and *Potamilus streckersoni* and pairwise *F*_ST_ between lineages derived from GBS data

Taxa (sample size)	1	2	3	4	AR	*H*_o_	*H*_e_	*F*_IS_
1. *P. amphichaenus* Neches (33)	—	1 (2,544)	2 (2,859)	340 (9,867)	1.742	0.168	0.251	0.314
2. *P. amphichaenus* Sabine (9)	0.041	—	4 (3,248)	492 (9,440)	1.705	0.161	0.228	0.249
3. *P. amphichaenus* Trinity (15)	0.110	0.096	—	490 (9,544)	1.669	0.162	0.228	0.258
4. *P. streckersoni* (34)	0.479	0.526	0.530	—	1.380	0.110	0.137	0.181

Note

*F*_ST_ values are reported on the lower triangle, and the number of fixed and private alleles is reported on the upper triangle. Acronyms are as follows: AR, allelic richness, *H*_o_, observed heterozygosity, *H*_e_, expected heterozygosity, and *F*_IS_, inbreeding coefficient (FIS).

**FIGURE 4:**
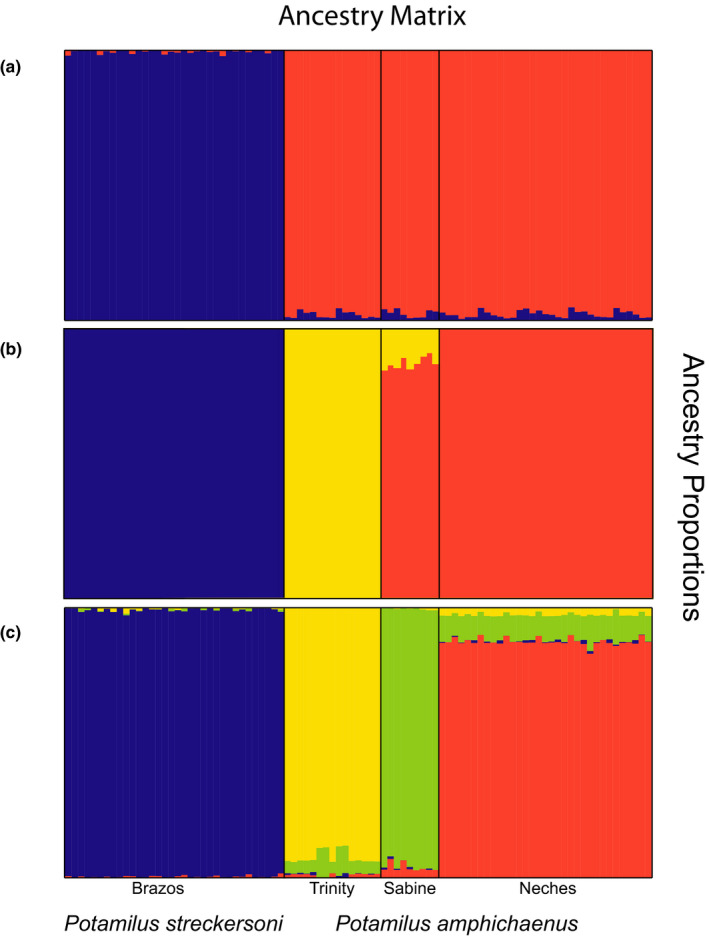
FastSTRUCTURE and TESS3 ancestry proportions for *Potamilus amphichaenus* and *Potamilus streckersoni* based on genotype‐by‐sequencing data: (a) TESS3 ancestry estimation for *K* = 2, (b) FastSTRUCTURE ancestry estimation for *K* = 3, and (c) TESS3 ancestry estimation for *K* = 4. Individuals are represented by a vertical line with coloration representing the admixture proportions from each population

**FIGURE 5:**
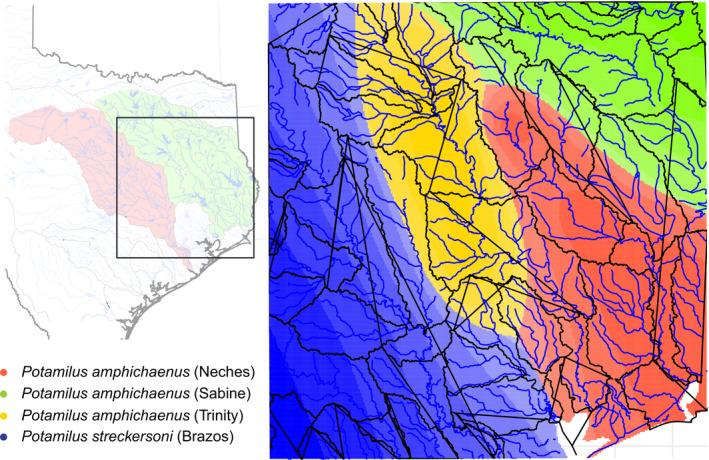
TESS3 distribution of genetic diversity based on *K* = 4 overlaid on hydrologic unit codes 8‐level. Colors correspond to the groups: *Potamilus amphichaenus* from the Neches—red, *P. amphichaenus* from the Sabine—green, *P. amphichaenus* from the Trinity—yellow, and *Potamilus streckersoni*—blue

Pairwise *F*_ST_ was much larger between *P. streckersoni* and all populations of *P. amphichaenus* than comparisons within *P. amphichaenus* populations (Table 2). Pairwise *F*_ST_ was also found to be larger between *P. amphichaenus* from the Trinity and *P. amphichaenus* from the Neches or Sabine (Table 2). The fastSTRUCTURE analysis using all individuals resolved *K* = 3 as the best value to explain structure and maximize likelihood in our GBS dataset: *P. amphichaenus* from the Sabine + *Neches*, *P. amphichaenus* from the Trinity, and *P. streckersoni* (Figure 4b). For the *P. amphichaenus* dataset, *K* = 2 (Sabine + *Neches*, Trinity) was resolved as the best value to maximize likelihood, while *K* = 3 (Sabine, Neches, Trinity) was supported as the best value to explain structure. For *P. streckersoni*, *K* = 1 was supported as the best value to explain structure and maximize likelihood. The TESS3 analysis using all data supported *K* = 2 selected by cross‐entropy plot, representing *P. amphichaenus* and *P. streckersoni* (Figure 4a). TESS3 analyses using species‐specific datasets supported *K* = 2 for *P. amphichaenus* (Sabine + *Neches*, Trinity) and *K* = 1 for *P. streckersoni*. The geographic distribution of *K* = 4 (Figure 4c), which aligned with the best *K* to explain structure in *P. amphichaenus* using model‐ and nonmodel‐based approaches, depicted genetic clusters spanning the drainages of capture: Sabine, Neches, Trinity, and Brazos (Figure 5). Convergence of path sampling analyses in SNAPP was supported by all steps having ESS values greater than 200. Bayes factor delimitation marginally supported *K* = 3 as the most likely clustering scenario and rejected *K* = 2, but BFD could not reject *K* = 4 (Table 3).

**TABLE 3:** Bayes factor delimitation based on path sampling analyses in SNAPP

Clusters	−ln	2lnBF	Reject
2	−10,252.51	**87.12**	**Yes**
3	−10,238.95	—	—
4	−10,241.01	4.12	No

Note

Bold 2lnBF values represent rejected clustering models.

Estimates of *N_e_* and *N_b_* for each group are reported in Table 4. Contrary to genetic diversity statistics, *N_b_* was higher in *P. streckersoni* (9.2) when compared to populations of *P. amphichaenus* (5.2–7.0). Estimates of *N_e_* were higher in *P. streckersoni* (5,243.7) when compared to *P. amphichaenus* from the Neches (538.3) but depicted wide confidence intervals (Table 4). Estimates of *N_e_* for *P. amphichaenus* from the Sabine and Trinity were infinite, presumably due to low sample size.

**TABLE 4:** Estimated values and jackknifed 95% confidence intervals for effective population size (*N_e_*) and effective number of breeders (*N_b_*) for *Potamilus amphichaenus* and *Potamilus streckersoni* derived from GBS data

Taxa (Drianage)	*N_e_*	*N_b_*
*P. amphichaenus* (Neches)	538.3 (173.3– Infinity)	5.5 (5.1–5.9)
*P. amphichaenus* (Sabine)	Infinity	5.2 (4.9–5.5)
*P. amphichaenus* (Trinity)	Infinity	7.0 (6.5–7.6)
*P. streckersoni* (Brazos)	5,243.7 (1,439.9–Infinity)	9.2 (8.3–10.1)

### 3.2 mtDNA analyses

**FIGURE 6:**
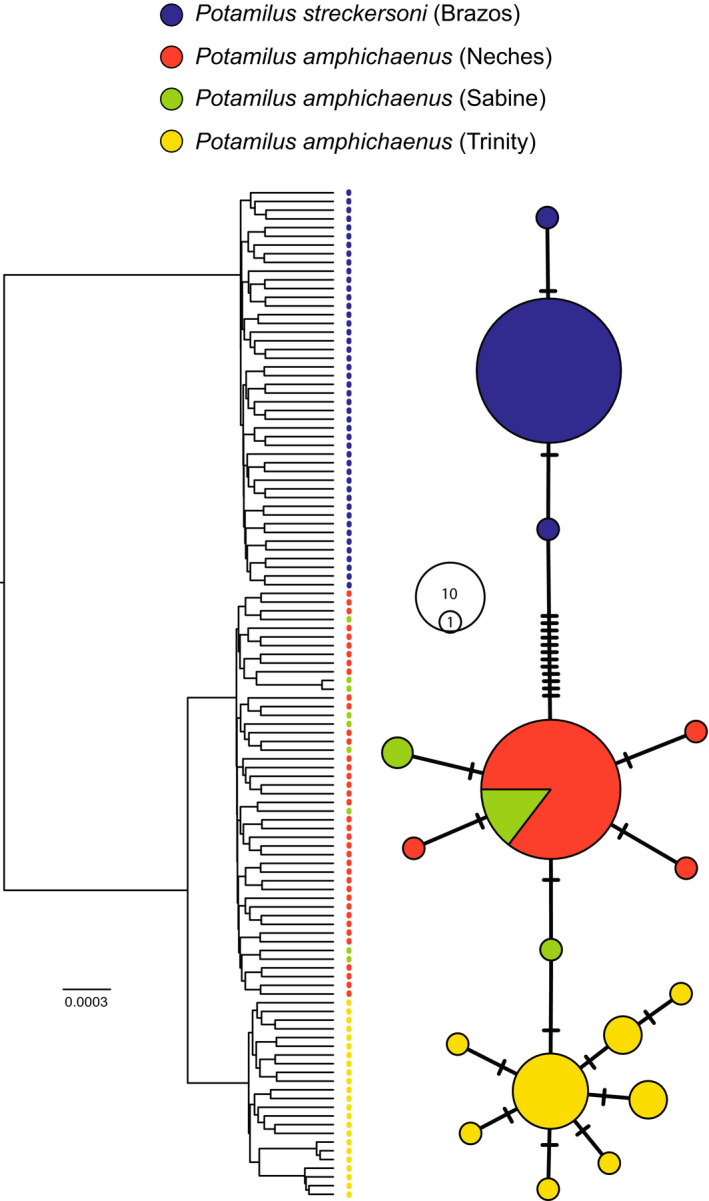
Phylogenetic reconstruction and haplotype network of *Potamilus amphichaenus* and *Potamilus streckersoni* based on ND1. Colors correspond to the following groupings: *P. amphichaenus* from the Neches—red, *P. amphichaenus* from the Sabine—green, *P. amphichaenus* from the Trinity—yellow, and *P. streckersoni*—blue. All major clades in the phylogenetic reconstruction were supported by posterior probability greater than 98. In the haplotype network, each circle represents a unique haplotype and size is relative to the number of individuals. Hash marks represent nucleotide substitutions

A total of 116 individuals were sequenced for ND1 (900 bp): *P. amphichaenus* from the Sabine (9), *P. amphichaenus* from the Neches (38), *P. amphichaenus* from the Trinity (23), and *P. streckersoni* (46). All ND1 sequences are deposited on GenBank, and accession numbers can be found in Table 1. The following substitution models were selected by bModelTest: ND1 1st codon position—TPM1 + I + G, ND1 2nd codon position—TrN, and ND1 3rd codon position—HKY + G. The ND1 reconstruction resolved three strongly supported clades representing *P. amphichaenus* from the Neches + Sabine, *P. amphichaenus* from the Trinity, and *P. streckersoni* (Figure 6). Convergence of the analysis was supported by ESS values for each parameter greater than 200, and a 10% burn‐in was deemed appropriate by Tracer.

Estimates for *h*, Hd, *k*, and *π* for each group are reported in Table 5. Estimates for all diversity statistics were larger in *P. amphichaenus* populations than in *P. streckersoni*, similar to GBS analyses. Genetic diversity was also greater in the Trinity and Sabine populations of *P. amphichaenus* when compared to the Neches population. Similar to GBS analyses, pairwise *F*_ST_ comparisons showed more divergence between (a) *P. streckersoni* and all populations of *P. amphichaenus*, and (b) *P. amphichaenus* from the Trinity and *P. amphichaenus* from the Neches or Sabine (Table 6). AMOVA depicted 97.39% of genetic variation was found among the four groups (i.e., *P. amphichaenus* from the Neches, *P. amphichaenus* from the Sabine, *P. amphichaenus* from the Trinity, and *P. streckersoni*), while 2.61% was found within groups. The haplotype network showed genetic differentiation of three groups similar to FastSTRUCTURE results: *P. amphichaenus* from the Sabine + Neches, *P. amphichaenus* from the Trinity, and *P. streckersoni* (Figure 6). There was limited divergence between each population of *P. amphichaenus* accompanied by haplotype sharing between the Sabine and Neches populations.

**TABLE 5:** Summary of genetic diversity statistics and pairwise *F*_ST_ for *Potamilus amphichaenus* and *Potamilus streckersoni* using mtDNA data

Taxa (sample size)	1	2	3	*H*	Hd	*k*	*π*
1. *P. amphichaenus* Neches (38)	—			4	0.154	0.158	0.00018
2. *P. amphichaenus* Sabine (9)	0.16587	—		3	0.556	0.611	0.00068
3. *P. amphichaenus* Trinity (23)	0.86819	0.76353	—	8	0.715	0.972	0.00110
4. *P. streckersoni* (46)	0.99260	0.97821	0.98973	3	0.086	0.087	0.00011

Note

Acronyms are as follows: *h*, unique haplotypes, Hd, haplotype diversity, *k*, mean number of nucleotide differences, and *π*, mean nucleotide diversity.

**TABLE 6:** Information regarding time periods used for distributional analyses, DPHS estimates for *Potamilus amphichaenus* and *Potamilus streckersoni* per time period, and the total number of surveys performed within each time period

Time period	Year range	*Potamilus amphichaenus*	*Potamilus streckersoni*	Total surveys
1	1901–1972	0.07	0.11	72
2	1973–1983	0	0.11	99
3	1984–1994	0.17	0.06	119
4	1995–1998	0.26	0.08	105
5	1999–2003	0.17	0.08	72
6	2004–2009	0.35	0.13	132
7	2010–2012	0.21	0.27	289
8	2013–2019	0.38	0.58	351

### 3.3 Distribution and abundance estimates

**FIGURE 7:**
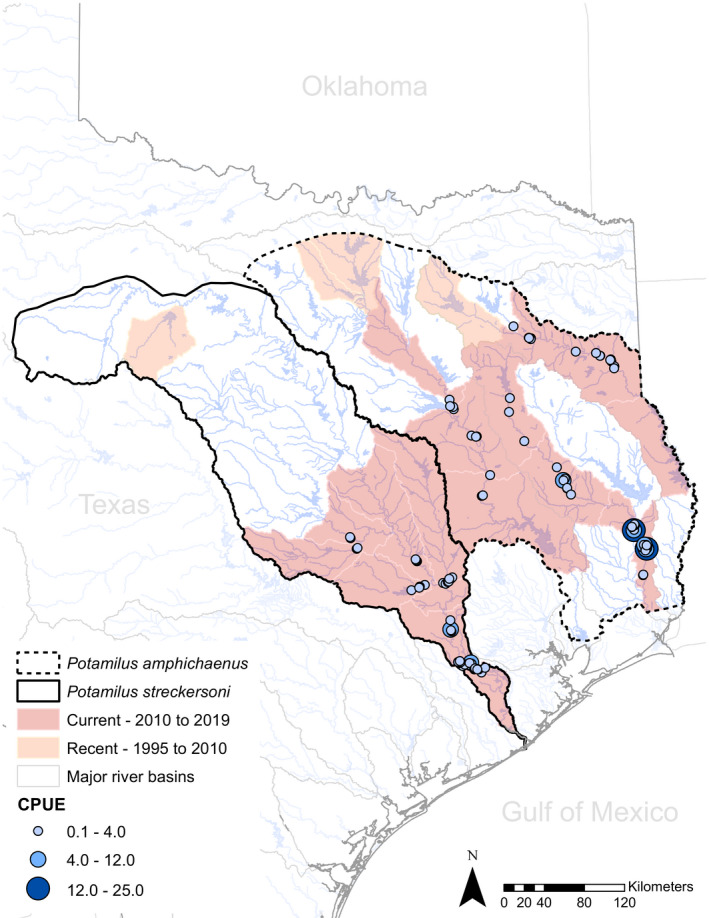
Geographical distribution and abundance estimates for *Potamilus amphichaenus* and *Potamilus streckersoni* throughout their respective ranges. The dashed and solid black lines denote the hypothetical historical distributions for *P. amphichaenus* and *P. streckersoni*, respectively. Hydrologic unit codes 8‐level are colored based on the most recent collection of live individuals. Variably sized and colored dots represent the relative abundance of each species as estimated by catch per unit effort (CPUE)

**FIGURE 8:**
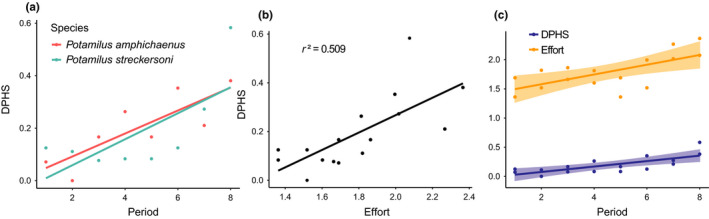
Summary of distributional analyses for *Potamilus amphichaenus* and *Potamilus streckersoni*: (a) Scatter plot and linear regression of detections per HUC sampled (DPHS) for *P. amphichaenus* (red) and *P. streckersoni* (blue) per time period, (b) scatter plot and linear regression analysis of DPHS and survey effort, (c) scatter plot and linear regression analysis for DPHS (blue) and survey effort (orange) per time period

We compiled information from 2,886 freshwater mussel surveys conducted in the Brazos, Neches, Sabine, and Trinity drainages for abundance and distributional analyses. Based on 106 surveys with adequate information for abundance estimation, CPUE estimates ranged from 0.13 to 25 for *Potamilus amphichaenus* (57 surveys) and 0.25 to 10.5 for *P. streckersoni* (49 surveys). *Potamilus amphichaenus* appears to be extirpated from the lower Sabine (downstream of Toledo Bend Reservoir) and lower Trinity drainages (downstream of Lake Livingston). In currently occupied reaches, *P. amphichaenus* is not abundant except for the lower Neches below Lake Steinhagen (Figure 7). *Potamilus streckersoni* appears to be extirpated from the Brazos River upstream of Lake Waco, but downstream of this reservoir remains moderately abundant and widely distributed (Figure 7). For DPHS, 1,239 surveys were suitable for analysis and collections spanned from 1901 to 2019. Live or fresh dead individuals of *P. amphichaenus* were detected in 96 surveys in 11 of the 24 total HUC8s sampled, and *P. streckersoni* was detected in 72 surveys in 8 of 16 total HUC8s sampled. DPHS estimates and the total number of surveys for each species per time period are reported in Table 6. Regressions depicted a general increasing trend in DPHS for each species with respect to time (Figure 8a); however, survey effort was shown to significantly influence DPHS (*r*² = 0.509; *p* = 0.001914) indicating that general increasing trends may be due to increased sampling effort rather than population expansion (Figure 8b,c).

## 4 DISCUSSION

Genetic data are effective at delineating clusters that can be used to inform management and restoration efforts, but our molecular analyses supported disputing number of genetic clusters within *P. amphichaenus* and *P. streckersoni* ranging from *K* = 2–4 (Figure 4). This is not unexpected because it is an unrealistic expectation that samples will conform to assumptions of each model and the notion of a “true” *K* may not be applicable to natural populations (e.g., Janes et al., 2017; Jombart et al., 2010; Pritchard et al., 2000; Raj et al., 2014). However, we do not see incongruence as an issue because ecological characteristics and life‐history information combined with molecular data can be used to identity appropriate spatial units for conservation (e.g., Funk et al., 2012; Palsbøll et al., 2007). Below, we outline the clustering scenarios supported by our molecular data, possible biogeographic, ecological, and life history‐driven explanations for differing clustering scenarios, a methodological approach to choose the “best” *K*, and how we integrate molecular findings and survey information to guide recovery planning for *P. amphichaenus* and *P. streckersoni*.

### 4.1 Assessing genetic structure in *Potamilus amphichaenus* and *Potamilus streckersoni*

The resolution of two genetic clusters (i.e., *K* = 2; Figure 4a) appears to be driven by species boundaries, with the two clusters representing *P. amphichaenus* and *P. streckersoni*. The bias toward clustering algorithms resolving *K* = 2 has been well discussed (e.g., Janes et al., 2017), and in our case, could be due to the fact that the majority of the variation in our molecular data could be explained by separating the two species (33.5%; Figure 3). Species boundaries have been shown to mask population structure within species when using model‐based approaches (e.g., Warner et al., 2015), and we addressed this issue by creating species‐specific datasets for *P. amphichaenus* and *P. streckersoni*. These analyses, along with traditional nonmodel‐based approaches, showed clear evidence for genetic structuring within *P. amphichaenus* that align with biogeographic hypotheses (Haag, 2010; Figures 2, 3, 4, 5, 6; Tables 2 and 4).

The resolution of three genetic clusters from GBS data (i.e., *K* = 3: *P. amphichaenus* from the Neches + Sabine, *P. amphichaenus* from the Trinity, and *P. streckersoni*) aligned well with results from mtDNA (Figures 4b and 6). Limited divergence between the Neches and Sabine is expected since the two drainages share an embayment, plus the two systems were likely interconnected during the last glacial maxima (Blum & Hattier‐Womack, 2009; Blum et al., 2013) leading to an increased possibility of recent gene flow. However, GBS data did show limited, but diagnosable, molecular differentiation between the Neches and Sabine systems, which supported the possibility of *K* = 4 (Figures 2, 3 and 4c; Table 2). Limited genetic divergence between the Neches and Sabine drainages could be reminiscent of repeated isolation and secondary contact, or also driven by human‐mediated extirpation events. Given similar phylogeographic patterns seen in congeners (Smith & Johnson, 2020) and life‐history characteristics, it is defensible that recent fluctuations in sea level likely led to limited differences at GBS markers rather than by human‐mediated extirpation events in the lower Sabine.

Several genetic clustering scenarios were resolved by our molecular methods, as outlined above. To address this issue, we used SNAPP to estimate the marginal likelihood of clustering scenarios resolved by molecular data. Although GBS data were able to diagnose the Neches and Sabine populations, BFD marginally supported three genetic clusters (Table 3): *P. amphichaenus* from the Neches + Sabine, *P. amphichaenus* from the Trinity, and *P. streckersoni*. We could not reject the model that separated the Neches and Sabine as independent clusters (*K* = 4; Table 3). Though our GBS dataset clearly contained more variability than our mtDNA dataset, BFD aligned with results supported by mtDNA and emphasizes the utility of mtDNA markers in investigating intraspecific relationships in freshwater mussels. Congruent patterns in mtDNA and nuclear markers may not hold across all freshwater mussel species (e.g., Chong et al., 2016), but our findings support that previous population genetic research using mtDNA should not be discredited.

Although GBS data were able to diagnose all independent river drainages, our data did not depict evidence of intradrainage population structure. Unlike most freshwater mussels, *Potamilus* species typically reach sexual maturity within 1 year and have a comparatively short lifespan (5–9 years) (Haag, 2012; Haag & Rypel, 2011), which theoretically would lead to an increased possibility of genetic drift. Despite these characteristics, our results are similar to other population genetic studies in freshwater mussels, where hypothetical genetic drift caused by dams constructed within the past 100 years was not detected (e.g., Elderkin et al., 2007; Hoffman et al., 2017). The lack of population structure intradrainage is not too surprising given the limited divergence exhibited between the Sabine and Neches drainages (Table 2), which have plausibly been separated since the last glacial maxima (Blum & Hattier‐Womack, 2009; Blum et al., 2013).

Freshwater mussels are nearly completely reliant on their host for dispersal (Haag, 2012; Vaughn, 2012), and patterns of genetic structuring can be heavily influenced by host use (e.g., Karlsson et al., 2014; Wacker et al., 2019). In the case of *P. amphichaenus* and *P. streckersoni*, both species are presumed to be host specialists, with glochidia only transforming on *A. grunniens* (Bosman et al., 2014; Smith et al., 2020), a wide ranging and mobile species that has been documented to travel over eighty kilometers in several days (Hansen et al., 2020) and up to several hundred kilometers during migration events (Funk, 1957). Dispersal capabilities of *A. grunniens* could therefore explain the lack of divergence within drainages, considering panmixia was likely historically present before impoundment of river systems. In addition to host use, other life‐history characteristics such as fecundity may also explain the lack of genetic structuring within drainages. Fecundity is often positively correlated with the retention of genetic diversity in natural populations (Ellegren & Galtier, 2016; Romiguier et al., 2014), and both *P. amphichaenus* and *P. streckersoni* have high annual fecundity (>1,000,000; Smith et al., 2020). In addition to a mobile host, high fecundity could be contributing to the lack of structuring within drainages, given the increased possibility of high recruitment and retention of neutral genetic variation.

### 4.2 Establishing conservation units for *Potamilus amphichaenus* and *Potamilus streckersoni*

The demand for water and hydropower, coupled with ongoing changing climate, has led to obvious reductions in streamflow, alteration of sediment transport, increased salinity levels, and exacerbated highly variable seasonal hydrological fluctuations (i.e., flood and drought) throughout the ranges of *P. amphichaenus* and *P. streckersoni* (Cañedo‐Argüelles et al., 2013; Philips et al., 2004; Randklev et al., 2011, 2013, 2017, 2019; Wellmeyer et al., 2005). These impacts combined with changes in land use have likely contributed to the widespread decline of both species throughout significant portions of their historical range (Ford et al., 2014, 2016; Howells et al., 1996; Randklev et al., 2011, 2017; Smith et al., 2019). Our estimates of abundance and contemporary distribution for *P. amphichaenus* and *P. streckersoni* support this observation (Figure 7), but DPHS estimates were less clear and depicted an increase in distribution relative to time for both species (Figure 8; Table 6). In recent years, however, there has been a resurgence of sampling effort that has resulted in rediscovery of presumed extirpated populations of numerous mussel species (e.g., Holcomb et al., 2015; Johnson et al., 2016; Randklev et al., 2010, 2012). We show a similar scenario within our focal species as survey effort significantly influenced DPHS (*p* = 0.001914), which leads us to hypothesize that the observed increasing trend in distribution for *P. amphichaenus* and *P. streckersoni* is a result of increased survey effort rather than range expansion.

The establishment of CUs within *P. amphichaenus* and *P. streckersoni* is the logical next step in recovery planning, and we found defensible evidence for the delineation of four genetically diagnosable CUs: *P. amphichaenus* from the Neches, *P. amphichaenus* from the Sabine, *P. amphichaenus* from the Trinity, and *P. streckersoni*. We do recognize that divergence between *P. amphichaenus* from the Neches and Sabine is limited (Table 2) and some analyses supported the two drainages as a single CU (Figures 4 and 6; Table 3); however, the genetic distinctiveness of the two drainages (Figures 2 and 3) warrants the recognition of independent CUs. Although the lack of mtDNA diagnosability has been used to justify mixing of CUs (Moritz, 1994), we caution the use of *P. amphichaenus* from the Neches in recovery efforts in the Sabine. The adaptive significance of the observed genetic distinctiveness between the Neches and Sabine is uncertain, but recovery efforts in these drainages should rely on stock sources within each CU to avoid genetic consequence. Our findings provide valuable information for natural resource managers, especially considering brood stock selection for recovery planning is likely less stringent than previously conceived.

Currently, populations of freshwater mussels for conservation and management practices are typically defined as geographic management units (GMUs), or a unit that is geographically or otherwise identifiable by man‐made and natural barriers (e.g., USFWS, 2018, 2020). In the case of *P. amphichaenus* and *P. streckersoni*, there is no molecular support for the subdivision of drainages into GMUs; however, we were unable to include material from multiple stream stretches where the species are presumed extirpated. The lack of molecular diagnosability does not discredit the use of GMUs for management and recovery practices because it is an unrealistic expectation that stressors and habitat suitability will be uniform throughout the range of freshwater mussel species (e.g., Randklev et al., 2019; Strayer et al., 2004; Vaughn & Taylor, 1999). Contemporary estimates of abundance for both species show stark contrasts with respect to geography (Figure 7), and rather than manage species at a drainage level, we encourage natural resource managers to integrate our CUs within existing frameworks. For example, population densities in the Neches drainage are variable (Figure 7), and translocation of *P. amphichaenus* from the lower Neches River (i.e., below B.A. Steinhagen Lake) to augment populations in the upper Neches River (i.e., above B.A. Steinhagen Lake) could be an effective recovery option to improve resiliency of the CU with limited genetic consequence. While CUs offer protection at the drainage level, delineation of GMUs within each CU will allow for more robust investigations of population characteristics such as relative abundance, age‐class structure, and threats to long‐term sustainability, all of which should be considered in recovery planning.

## 5 CONCLUSION

Our study represents a model for population genomic assessments of freshwater mussels and provides information for natural resource managers in the development of conservation and recovery strategies for *P. amphichaenus* and *P. streckersoni*. Given the wide diversity of host use in freshwater mussels, it is an unrealistic expectation that other imperiled species that co‐occur with *P. amphichaenus* and *P. streckersoni* will depict similar patterns of genetic structuring. Thus, there remains a critical need for robust molecular investigations to support recovery planning for many imperiled species. As genomic resources are developed, the identification of potentially adaptive loci through RNA sequencing and whole genome resequencing may improve brood stock selection and species recovery.

## CONFLICT OF INTEREST

None declared.

## AUTHOR CONTRIBUTIONS

**Chase H. Smith:** Conceptualization (lead); Data curation (lead); Formal analysis (lead); Investigation (lead); Visualization (lead); Writing‐original draft (lead). **Nathan A. Johnson:** Conceptualization (supporting); Resources (equal); Writing‐review & editing (equal). **Clinton R. Robertson:** Resources (equal); Writing‐review & editing (equal). **Robert D. Doyle:** Funding acquisition (lead). **Charles R. Randklev:** Conceptualization (supporting); Funding acquisition (lead); Resources (equal); Writing‐review & editing (equal).

## Data Availability

All data and materials in this study are freely available in data repositories. Raw reads are deposited in the SRA (BioProject ID: PRJNA663379), and sample accession numbers can be found in Table 1. Raw SNP calls and R scripts used for data processing are available on GitHub (https://github.com/chasesmith15/Potamilus_EE). ND1 sequences are deposited on GenBank, and accession numbers can be found in Table 1. Survey data used in this study are available in the Mussels of Texas database (https://mussels.nri.tamu.edu/).
